# Completing the hybridization triangle: the inheritance of genetic incompatibilities during homoploid hybrid speciation in ragworts (*Senecio*)

**DOI:** 10.1093/aobpla/ply078

**Published:** 2019-01-06

**Authors:** Adrian C Brennan, Simon J Hiscock, Richard J Abbott

**Affiliations:** 1 Department of Biosciences, University of Durham, South Road, Durham, UK; 2 School of Biology, University of St Andrews, St Andrews, Fife, UK; 3 Department of Plant Sciences, University of Oxford, Oxford, UK

**Keywords:** Genetic incompatibility, genetic mapping, genomic rearrangement, reproductive isolation, transmission ratio distortion

## Abstract

A new homoploid hybrid lineage needs to establish a degree of reproductive isolation from its parent species if it is to persist as an independent entity, but the role hybridization plays in this process is known in only a handful of cases. The homoploid hybrid ragwort species, *Senecio squalidus* (Oxford ragwort), originated following the introduction of hybrid plants to the UK approximately 320 years ago. The source of the hybrid plants was from a naturally occurring hybrid zone between *S. aethnensis* and *S. chrysanthemifolius* on Mount Etna, Sicily. Previous studies of the parent species found evidence for multiple incompatibility loci causing transmission ratio distortion of genetic markers in their hybrid progeny. This study closes the hybridization triangle by reporting a genetic mapping analysis of the remaining two paired cross combinations between *S. squalidus* and its parents. Genetic maps produced from F_2_ mapping families were generally collinear but with half of the linkage groups showing evidence of genomic reorganization between genetic maps. The new maps produced from crosses between *S. squalidus* and each parent showed multiple incompatibility loci distributed across the genome, some of which co-locate with previously reported incompatibility loci between the parents. These findings suggest that this young homoploid hybrid species has inherited a unique combination of genomic rearrangements and incompatibilities from its parents that contribute to its reproductive isolation.

## Teaser

A genetic mapping approach was taken to study the inheritance of genetic incompatibilities between a new homoploid hybrid species, *Senecio squalidus*, and its two progenitor species. Multiple genomic regions, including genomic rearrangements, were found to contribute to hybrid incompatibility between these species. In particular, the new hybrid species had inherited a mixture of genetic incompatibilities against both parents. These results supported the hypothesis that re-assortment of pre-existing parental genetic incompatibilities in hybrids can contribute to reproductive isolation between hybrids and their progenitors, potentially leading to further divergent evolution.

## Introduction

Hybridization is an important contributor to biodiversity and speciation with approximately 25 % of all plant species and 10 % of all animal species estimated to have experienced hybridization during their evolution (Mallet 2005; Baack and Reiseberg 2007). Beyond introgression of novel genetic diversity, the contribution of hybridization to speciation is of particular interest. Genome duplication following hybridization (allopolyploidy) is a frequent mode of speciation in plants with increasing evidence for its occurrence in animals (Rieseberg and Willis 2007; Mable *et al.* 2011; Soltis *et al.* 2014). Despite the challenges involved in identifying and confirming cases of hybrid speciation without change in ploidy, i.e. homoploid hybrid speciation, improving genetic technologies are accelerating the rate of identification of examples of homoploid hybrid species (Abbott *et al.* 2013; Yakimowski and Rieseberg 2014; Goulet *et al.* 2017). Moreover, textbook examples of homoploid hybrid speciation are now available for *Heliconius* butterflies (Mavarez *et al.* 2006; Duenez-Guzman *et al.* 2009) and *Helianthus* sunflowers (Rieseberg 2001; Burke *et al.* 2004; Lai *et al.* 2005), although proof of homoploid hybrid speciation remains controversial (Schumer *et al.* 2014b; Nieto Feliner *et al.* 2017).

Homoploid hybrid speciation is theoretically challenging to explain, particularly when hybrids show sympatry with their progenitors, because reproductive barriers will usually be incomplete, and ongoing hybridization and gene flow are expected to limit the evolution of reproductive isolation and the origin of a homoploid hybrid species (Abbott *et al.* 2013; Schumer *et al.* 2014b). However, various evolutionary processes have been identified as contributing to hybrid speciation without ploidal change. Hybridization is effective at generating a range of new trait combinations and transgressive trait expression that occasionally enable hybrids to exhibit higher fitness than parents in particular ecological contexts (Buerkle *et al.* 2000; Lexer *et al.* 2003; Schwarz *et al.* 2005; Jiggins *et al.* 2008; Stelkens and Seehausen 2009). Under these conditions, positive selection can promote the establishment and persistence of new homoploid hybrid species even in the presence of ongoing gene flow (Buerkle *et al.* 2000). This process is further facilitated if novel hybrid traits, such as changes in reproductive phenology, pollinator or mating preference, directly reduce gene flow between parents and their hybrids (Servedio *et al.* 2011; Marques *et al.* 2016).

Another mechanism reducing gene flow and promoting reproductive isolation between hybrids and their progenitors concerns genetic incompatibilities caused by chromosomal rearrangements and/or negative epistasis between parental alleles in the hybrid. New hybrids will often show reduced fitness due to (1) meiotic problems caused by possession of different parental chromosomal rearrangements and (2) the negative interaction of new combinations of alleles inherited from both parents. However, fitness can be recovered through re-assortment of both chromosomal rearrangements and genetic incompatibilities in later generation hybrids (Grant 1981; McCarthy *et al.* 1995; Lai *et al.* 2005; Schumer *et al.* 2015).

Although genetic incompatibilities might be of individually small effect, they appear to be common and their combined effects can be potent evolutionary drivers of speciation (Orr and Presgraves 2000; Presgraves 2010; Schumer *et al.* 2014a; Lindtke and Buerkle 2015). For example, alleles at different loci that have negative epistatic interactions (Bateson–Dobzhansky–Muller incompatibilities or BDMs) arise readily between isolated populations either through divergent selection or through genetic drift (Coyne and Orr 2004; Paixão *et al.* 2014; Schumer *et al.* 2015). Recent theoretical models further suggest that selection against negative epistasis within hybrid populations can lead to fixation of combinations of alleles that show genetic incompatibility between hybrids and both parents, thus promoting reproductive isolation (Paixão *et al.* 2014; Schumer *et al.* 2015). In reality, multiple interacting evolutionary processes probably interact to promote homoploid hybrid speciation. For example, hybrid *Helianthus* species clearly show contributions from both hybridization-induced genomic reorganization and adaptation to novel ecological niches (Rieseberg 2001; Lexer *et al.* 2003; Burke *et al.* 2004; Lai *et al.* 2005).

Genetic mapping studies are effective at giving a genome-wide perspective of the evolutionary processes driving homoploid hybrid speciation. This study returns to the recent homoploid hybrid origin of *S. squalidus* (Oxford ragwort) from its parental species, *S. aethnensis* and *S. chrysanthemifolius*. The human-aided translocation of hybrids to the UK from a natural hybrid zone on Mount Etna, Sicily, approximately 320 years ago was crucial to the origin of *S. squalidus* as a new stabilized hybrid species, and its subsequent invasive spread (Abbott *et al.* 2009). While geographical isolation allowed the establishment of this homoploid hybrid species, the contribution of genetic incompatibilities to reproductive isolation at the early stages of hybrid speciation is still of interest.

*Senecio squalidus* shows molecular genetic and quantitative trait divergence from each parent species (and hybrids occurring on Mount Etna), as well as local adaptation associated with latitude within the UK and between the UK and Sicily (Allan and Pannell 2009; Brennan *et al.* 2012; Ross 2010). The hybrid zone on Mount Etna is stable despite relatively high gene flow between parent species since they first diverged during the last 150k years (Filatov *et al.* 2016) due to both intrinsic hybrid incompatibilities and strong divergent ecological selection associated with elevation (Brennan *et al.* 2009; Ross *et al.* 2012; Chapman *et al.* 2013, 2016; Brennan *et al.* 2014). All three species are readily hybridized in the glasshouse with few apparent fertility or fitness consequences apart from low seed germination at the F_3_ generation (Hegarty *et al.* 2008; Brennan *et al.* 2013). However, recent genetic mapping studies using F_2_ families derived from crosses between *S. aethnensis* and *S. chrysanthemifolius* have characterized genetic incompatibilities in the form of transmission ratio distortion (TRD), breakdown of fitness at the F_2_ generation, and associations between transmission ratio distortion loci (TRDL) and quantitative traits (Chapman *et al.* 2013, 2016; Brennan *et al.* 2014, 2016). These characteristics of hybrid crosses function as genetic incompatibilities by increasing reproductive barriers between taxa (Hall and Willis 2005; Moyle and Graham 2006). Here, we conduct, for the first time, genetic mapping studies using F_2_ crosses between each of these two species and their hybrid descendent, *S. squalidus*. In contrast to the hybrid zone on Mount Etna, where selection against hybrids prevents individual hybrid lineages from persisting for many generations (Brennan *et al.* 2009), *S. squalidus* in the UK is a stabilized hybrid descendent that has adapted to a new environment (Abbott *et al.* 2009). This set of hybridizing taxa is therefore of considerable interest for a better understanding of the early stages of hybrid speciation.

The genetic mapping studies reported here complete the hybridization triangle of all paired cross combinations between the two parents and their hybrid derivative, and thus increase our understanding of hybrid evolution in this system. Specifically, we test the following hypotheses based on prior knowledge about this hybridizing *Senecio* system and predictions from the hybrid speciation literature. (1) Hybrid speciation has been accompanied by little major genomic restructuring as suggested by similar genome structures of the parent species; (2) intrinsic genetic incompatibilities are present between *S. squalidus* and its parents; and (3) they are likely to have been inherited in *S. squalidus* from its parent species. We discuss our findings in terms of their wider implications for understanding hybridization and homoploid hybrid speciation.

## Material and Methods

### Mapping families

F_2_ mapping families were founded from each of three paired cross combinations between three glasshouse grown (F_0_) individuals representing each of *S. aethnensis*, *S. chrysanthemifolius*, and *S. squalidus*. *Senecio aethnensis* and *S. chrysanthemifolius* F_0_ parental individuals were originally sampled as seed from populations VB and C1 on Mount Etna as described in James and Abbott (2005), whereas the *S. squalidus* F_0_ individual was sampled as seed from the Oxford (Ox), UK, population as described in Hiscock (2000). Reciprocal controlled crosses were performed between parental individuals by gently brushing together open flower heads and excluding illegitimate pollen transfer with pollination bags before and after pollination as described in Hiscock (2000) to produce F_1_ families where the maternal and paternal species of each individual were known. Floral emasculations have been shown to be unnecessary for these typically strongly self-incompatible species (Hiscock 2000). Seeds resulting from these crosses were grown to flowering stage and further reciprocal crosses were performed between full-sib F_1_ individuals with distinct maternal cytoplasms (i.e. F_1_ progeny of the same parental individuals but produced from opposite cross directions). From each of the three originally paired species combinations, one reciprocally crossed pair of F_1_^’^s was chosen to found each of three F_2_ mapping families, maintaining approximately equal frequencies of maternal cytoplasm per family. The family hereafter referred to as F2AC was derived from the original cross between *S. aethnensis* and *S. chrysanthemifolius*, F2AS was derived from the cross between *S. aethnensis* and *S. squalidus*, and F2CS was derived from the cross between *S. chrysanthemifolius* and *S. squalidus*. The F2 individuals were labelled in a way to keep track of their maternal origin. The analysis of the F2AC family was previously described in Brennan *et al.* (2014) but is included here for completeness and comparison with the analysis of the two other F2 families.

### Genotyping

Genomic DNA was extracted from leaf tissue samples as described in Brennan *et al.* (2009) from all F_0_, F_1_ and F_2_ plants. The number of F_2_ offspring from which DNA was extracted was 100, 100 and 107 for the F2AC, F2AS and F2CS mapping families, respectively. Approximately 10 % of samples were extracted twice to serve as quality controls.

Samples were genotyped for eight selective primer combinations of Amplified Fragment Length Polymorphisms (AFLPs) according to Brennan *et al.* (2014), with the final choice of primer combinations and bands scored based on polymorphism in the F0 and F1 parents, high scorability (fluorescence signal >100 rfu), and high repeatability (repeated samples >95 % similar). In addition, the F_2_ mapping families were genotyped for a total of 75 codominant genetic markers that were found to be polymorphic in the F_0_ and F_1_ parents. These comprised 61 expressed sequence tag simple sequence repeats (EST SSRs) and EST indels that were developed from the *Senecio* expressed sequence tag database (www.seneciodb.org, Hegarty *et al.* 2008), and 14 other codominant SSRs and indels derived from previously published *Senecio* sequences as described in Brennan *et al.* (2014). Genetic markers were amplified using a three primer system with universal M13 primers fluorescently labelled with FAM6, HEX or NED using a common Polymerase Chain Reaction (PCR) protocol for genotyping on a Beckman Coulter CEQ 8000 capillary sequencer as described in Brennan *et al.* (2009).

### Genetic mapping

Genetic maps were constructed for each of the mapping families using JoinMap v4.0 (Van Ooijen 2001) as previously described for the F2AC mapping family (Brennan *et al.* 2014). Because each F_2_ mapping family was derived from two outbred F_0_ parents, between two and four alleles segregated at each polymorphic genetic locus. Genotype data for AFLPs and other genetic markers were first formatted according to JoinMap outcrossed mapping family (Crossed Parent or CP type) that allows markers with different segregation patterns, phases and dominance expression to be combined for genetic map construction. Linkage groups (LGs) were identified as sets of markers sharing at least one logarithm of odds (LOD) linkage score >3 and genetic distances <20 Kosambi centiMorgans (cM) following LG regression mapping. Marker order for each LG was determined by iterative rounds of regression mapping, excluding markers that had a large influence on marker order or goodness-of-fit statistics. Each genetic map was summarized for total length according to two different estimators (Chakravarti *et al.* 1991, Fishman *et al.* 2001), map coverage according to Fishman *et al.* (2001), and genetic marker clustering using dispersion tests (Brennan *et al.* 2014).

### Genomic rearrangements

Equivalent LGs in each of the three genetic maps were identified on the basis of shared genetic markers. A few genetic markers were found to be mapped to non-equivalent LGs, possibly representing genomic translocations. These markers were excluded from subsequent analyses of relative marker order (synteny). The relative orientation of equivalent LGs on different genetic maps with five or more shared markers was identified by comparing the results of Kendall’s tau correlation tests of shared genetic marker rank order in R v3.1.2 (R Development Core Team 2017). The relative orientation of LGs with fewer than five shared markers was determined by comparing the mean absolute difference in marker rank order. Overall synteny of genetic maps was then assessed using Kendall’s tau correlation tests. Potential genomic rearrangements were identified by examining marker order differences for each paired LG comparison where no combination of LG orientations across the three maps could counteract uncorrelated marker orders in at least one map. Rearrangments typically manifested as transversions with the sequence of marker order differences switching from positive to negative or vice versa. The start and end points of rearrangements were estimated as half the distance between the outermost rearranged marker and the next non-rearranged marker. Recombination rates were compared between rearranged and non-rearranged genomic regions in each map using two sample unpaired rank-sum Wilcox tests of mapped marker distances.

### Transmission ratio distortion

To identify genomic regions containing genetic incompatibilities between species, genotype data for each marker in each mapping family were initially tested (Test 1) for TRD against the null hypothesis of Mendelian segregation with χ^2^ tests using Microsoft Excel 2010 (Microsoft Corp). Genetic markers were considered to show TRD if χ^2^ tests were significant at a 95 % confidence level before examining the effect of using a 100-fold stricter confidence level to account for multiple marker tests per map.

All markers linked to a genetic incompatibility locus are expected to show similar patterns of TRD. Therefore, clusters of genetic markers showing similar patterns of TRD and linked by less than 10 cM map distance were identified as TRDLs. Single genetic markers showing independent patterns of TRD and located more than 10 cM from other markers with TRD were considered as additional TRDLs, albeit with less supporting evidence. The extent of a genomic region affected by TRD was estimated as halfway to the next marker not showing TRD.

Genotype data were further examined to determine the possible causes of TRD by means of the following tests. Test 2: Cytonuclear incompatibilities dependent on cross direction were examined by testing for genotypic TRD in the subsets of each mapping family in each reciprocal cross direction (i.e. sharing the same species cytoplasm). Test 3: For those TRDLs containing genetic markers for which all alleles could be assigned to each F_0_ parent, the influence of pre-zygotic incompatibilities acting at the haploid gametophyte stage was assessed by performing χ^2^ tests of allelic frequencies against null expectations. Dominantly scored markers or markers where parental genotypes shared some alleles could not be tested in this way. Test 4: Also, for those TRDLs containing genetic markers for which all alleles could be assigned to each F_0_ parent, deficits or excesses of heterozygotes in terms of F_0_ parental origin were tested with χ^2^ tests against null expectations. Test 5: Two-locus negative epistatic interactions, also known as BDMs were tested by building contingency tables of genotype combinations for the most distorted marker within each pair of TRDLs and performing Fisher’s exact tests for biases in genotype frequency combinations.

### Co-location of TRDLs across the different genetic maps and with genomic rearrangements

The locations of observed TRDLs and genomic rearrangements were compared between the different genetic maps by transposing them onto the F2AC map according to common genetic markers. For this, the location of the genetic marker in the F2AC map that showed most TRD in each TRDL and the midpoint of each rearranged genomic region were used for subsequent analyses of co-location. Co-location between TRDLs identified by genotype tests in each pair of genetic maps after transposition onto the F2AC genetic map was tested using sampling without replacement tests as described in Brennan *et al.* (2016). Briefly, the genetic map was divided into ‘n’ intervals of equal length and the frequency of TRDL occurrence and co-occurrence in each interval was tested against null expectations of no association between the genomic distributions of TRDLs in each genetic map. The influence of including either TRDs with individual- and map-level 95 % confidence levels at a range of interval sizes on the test statistic was investigated. Those TRDLs that mapped to different equivalent LGs when transposed onto the F2AC genetic map were excluded from the TRDL co-location analysis due to uncertainty over their map position. Following these analyses, the co-location between genomic rearrangements and TRDLs was examined in the same way.

## Results

### Genetic maps

**Figure 1.**
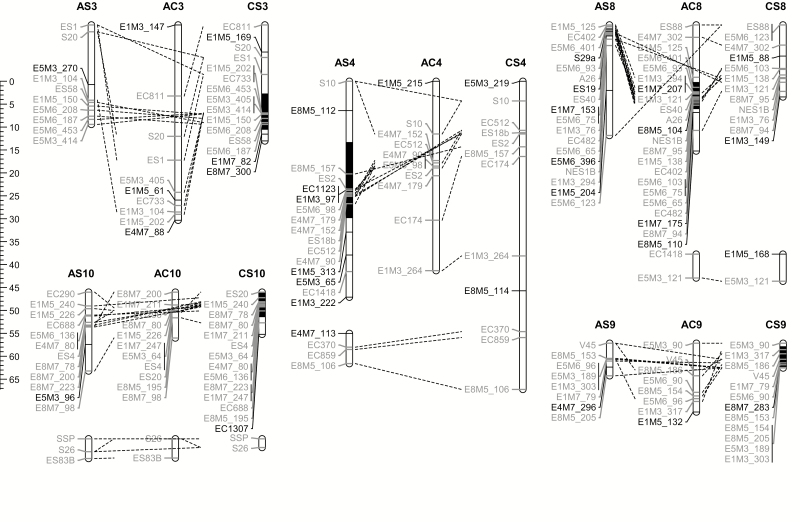
Interleaved genetic maps of selected linkage groups (LGs) from the F2AC, F2AS and F2CS mapping families. Map distances in Kosambi centiMorgans are shown in the scale to the left of LGs. Linkage groups are represented by vertical bars with mapped marker positions indicated with horizontal lines. Linkage group names are presented in bold above each LG with letters indicating the pair of F_0_ species; *S. aethnensis* (A), *S. chrysanthemifolius* (C) or *S. squalidus* (S) that founded the mapping family and numbers indicating equivalent LGs that share genetic markers across the three maps. Weakly linked LGs that are thought to belong to the same chromosome are aligned vertically under a single overall LG name. Marker names are listed to the left of LGs in grey if they are common to another genetic map or in black if they are uniquely present on that genetic map. Dotted lines link common marker positions on the equivalent LGs of different genetic maps. Black shaded portions of LGs indicate chromosomal transversions identified from switches in marker order compared with equivalent LGs. See **Supporting Information—Figure S1** for a depiction of all LGs corresponding to 10 chromosomes.

Details of all genetic markers used to genotype individuals for the construction of genetic maps including: type (SSR, indel or AFLP), EST match in the *Senecio* EST database, primer sequences, F_0_ genotypes and genetic mapping information are provided in **Supporting Information—Table S1**. The full F2AS, F2AC and F2CS genetic maps are illustrated in **Supporting Information—Figure S1** with a summary of five LGs presented in Fig. 1 and summary statistics provided in Table 1. The F2AC genetic map was described previously (see Brennan *et al.* 2014 and Dryad Digital Repository: doi:10.5061/dryad.7b56k) and is included here for comparative purposes. F_2_ genotype data used to construct the F2AS and F2CS genetic maps are available at (DRYAD Digital Repository; doi:10.5061/dryad.82d5f33).

**Table 1.** Summary genetic linkage map statistics for the F2AC, F2AS and F2CS genetic maps

Linkage group	Length			No. of genetic markers			No. of codominant markers			No. dominant markers			Add 2S length			Method 4 length		
	F2AC	F2AS	F2CS	F2AC	F2AS	F2CS	F2AC	F2AS	F2CS	F2AC	F2AS	F2CS	F2AC	F2AS	F2CS	F2AC	F2AS	F2CS
1	44.5	47.0	46.4	18	18	18	14	11	12	4	7	6	50.1	51.8	51.0	49.7	52.5	51.9
2	29.1	46.5	39.1	7	14	12	3	4	5	4	10	7	34.7	51.3	43.7	38.8	53.7	46.2
3	42.6	21.7	25.3	10	10	15	4	3	5	6	7	10	48.2	26.5	29.9	52.1	26.5	28.9
4A	41.3	39.9	67.2	10	14	12	4	5	7	6	9	5	46.9	44.7	71.8	50.5	46.0	79.4
4B	–	6.6	–	–	4	–	–	2	–	–	2	–	–	11.4	–	–	11.0	–
5A	25.8	26.9	23.0	9	7	12	4	3	5	5	4	7	15.1	31.7	27.6	15.8	35.9	27.2
5B	9.5	13.2	6.7	4	3	2	4	3	2	0	0	0	47.3	18	11.3	52.1	26.4	20.1
6A	41.7	28.3	21.0	9	16	5	2	5	0	7	11	5	19.8	33.1	25.6	16.6	32.1	31.5
6B	–	–	8.9	–	–	8	–	–	5	–	–	3	–	–	13.5	–	–	11.4
7A	14.2	12.3	15.9	13	10	15	1	2	1	12	8	14	19.8	17.1	20.5	16.6	15.0	18.2
7B	3.2	–	3.2	2	–	2	1	–	1	1	–	1	8.8	–	7.8	9.6	–	9.6
8A	27.5	24.1	15.7	22	18	12	6	7	2	16	11	10	33.1	28.9	20.3	30.1	26.9	18.6
8B	5.2	–	5.9	2	–	2	1	–	0	1	–	2	10.8	–	10.5	15.6	–	17.7
9	15.0	7.1	5.4	8	8	12	1	1	1	7	7	11	20.6	11.9	10.0	19.3	9.1	6.4
10A	10.0	11.4	9.2	11	11	14	3	3	4	8	8	10	15.6	16.2	13.8	12.0	13.7	10.6
10B	4.2	4.3	1.9	2	4	2	2	3	2	0	1	0	9.8	9.1	6.5	12.6	7.2	5.7
total	313.8	289.3	294.8	127	137	143	50	52	52	77	85	91	391.6	351.2	364.3	407.1	356.0	383.3
mean	22.41	22.25	19.65	9.07	10.54	9.53	3.57	4.00	3.47	5.50	6.54	6.07	27.97	27.01	24.28	29.08	27.39	25.56
stdev	15.56	14.91	18.52	5.88	5.22	5.55	3.37	2.61	3.20	4.55	3.69	4.33	15.56	14.91	18.52	16.65	16.12	20.18
unmapped unlinked				9	6	4	1	2	2	8	4	2						
unmapped problematic				9	8	9	3	2	1	6	6	8						
unmapped total				18	14	13	4	4	3	14	10	10						

Linkage groups are named as in Brennan *et al.* (2014) where ‘A’ and ‘B’ after numbers indicate that these LGs probably belong to the same chromosome. ‘–’ indicates that an equivalent linkage was not observed in a genetic map; this is sometimes due to mapping of these markers to the preceding LG. Map length measures are in Kosambi centiMorgan units. Add 2S length is an estimate of chromosome length calculated as LG length plus twice mean LG distance. Method 4 length is another estimate of chromosome length calculated as LG length times (marker number + 1)/(marker number − 1).

Each map comprised 13 to 15 LGs based on our minimum linkage criteria of greater than 3 LOD linkage between pairs of markers and <20 Kosambi cM distance between markers. However, some pairs of LGs showed weaker evidence of linkage or were part of the same equivalent LG in another genetic map leading to the conclusion that these maps correspond to the *n* = 10 chromosomes expected for these *Senecio* species (Alexander 1979). The F2AS and F2CS maps contained slightly more markers (139 and 143, respectively) than the F2AC map (127 markers), but were slightly shorter in total map length (289.3 and 294.8 Kosambi cM, respectively) relative to the F2AC map (313.8 Kosambi cM). Genetic markers were separated by mean distances of 2.8, 2.4 and 2.3 Kosambi cM within the F2AC, F2AS and F2CS genetic maps, respectively, with between >95.6 % and > 99.8 % of the genome predicted to be within 5 and 10 Kosambi cM of a mapped maker. However, according to dispersion tests the distributions of genetic markers were significantly clumped across all three maps (*P* < 10^–16^, all maps), indicating that some regions of each map show better marker coverage than others.

### Genomic rearrangements

Paired comparisons between the three genetic maps showed that almost half (62 to 70) of the genetic markers were shared between each pair of maps allowing identification and orientation of equivalent LGs **[see****Supporting Information—Figure S1** and Table 2**]**. The maps showed high synteny overall as indicated by high Kendall’s tau correlation coefficients for genetic marker rank order (Fig. 2). Based on the magnitude of the tau coefficient, the overall AS-CS comparison showed highest synteny, followed by AC-AS and then AC-CS (Fig. 2). Nonetheless, one to three genetic markers per map were discordant with the overall sharing of markers between LGs and were present on a non-equivalent LG in another of the maps, as labelled in Fig. 2. Moreover, ten instances of switches in marker order, corresponding to five genomic regions, were detected when shared marker order was examined between pairs of maps at an LG level (Fig. 1, Table 2). These likely genomic rearrangements were present on LGs 8 and 4 in the F2AC and F2AS maps, respectively, and on LGs 3, 9 and 10 in the F2CS map, as highlighted in Fig. 1. It was further evident that mapped marker distances were significantly shorter within genomic regions associated with these rearrangements compared with other genomic regions (mean ± SD 0.6 ± 0.7 versus 3.8 ± 4.1 for F2AC, 0.6 ± 0.8 versus 3.1 ± 3.8 for F2AS, 0.5 ± 0.6 versus 3.0 ± 4.3 for F2CS, Wilcox test *P* < 10^–05^ for each map) in accordance with expectations of reduced recombination within rearranged regions.

**Table 2.** Summary of transversions detected by tests for synteny of shared marker order for each LG in each paired genetic map comparison.

Map comparison	# markers	Tau (*P* value)	absdiff- abs(diff)	AC LG	AC start marker	AC start position (cM)	AC end marker	AC end position (cM)	length (cM)	transverted map	min start position (cM)	max end position (cM)	length (cM)
F2AC and F2CS	6	0.69 (0.06)	0.67	AC3	E5M3_405	36.5	E1M5_202	41.3	4.8	F2CS	34.2	41.3	7.1
F2AS and F2CS	8	0.21 (0.55)	2.25	AC3	E5M6_187	34.2	E5M6_453	36.4	2.2				
F2AS and F2CS	7	0.1 (0.76)	2.29	AC4	E4M7_152	17.3	E4M7_179	20.6	3.3	F2AS	16.1	20.6	4.5
F2AS and F2CS	8	0.57 (0.06)	1	AC4	E8M5_157	16.1	EC512	18.1	2				
F2AS and F2CS	11	0.38 (0.12)	2.36	AC8	E5M6_401	13.6	EC482	17.5	3.9	F2AC	13.6	17.5	3.9
F2AC and F2CS	9	0.56 (0.04)	1.33	AC8	E1M3_121	14.8	E5M6_103	16.6	1.8				
F2AC and F2CS	6	0.33 (0.47)	1.33	AC9	V45	5.8	E1M3_317	12.7	6.9	F2CS	5.8	15.7	9.9
F2AS and F2CS	6	0.46 (0.22)	1	AC9	E8M5_153	12.3	E8M5_205	15.7	3.4				
F2AC and F2CS	8	0.49 (0.10)	1.25	AC10	E1M7_211	2.8	ES20	4.4	1.6	F2CS	2.8	5.4	2.6
F2AS and F2CS	9	0.43 (0.11)	1.56	AC10	E8M7_223	5.1	EC688	5.4	0.3				

# markers is the number of common markers on the equivalent LG of each pair of compared genetic maps. tau is the Kendall paired rank correlation test summary statistic, values range from 0 to 1 with larger values indicating higher synteny. *P* value is the probability of the observed tau values, test results greater than 0.05 are shown indicating that the compared marker orders are insignificantly different than random. absdiff-abs(diff) summarizes marker order differences, it is the mean marker absolute difference in rank order minus the absolute of mean marker differences in rank order, larger absdiff-abs(diff) values indicate transversions in marker order that start with negative differences and end with positive difference that cancel each other out leading to smaller abs(diff) values. AC LG is the equivalent F2AC LG where the difference in marker order was found. AC start/end marker is the nearest F2AC mapped marker to the start/end of the change in marker order. AC start/end position is the equivalent F2AC map start/end position of the change in marker order, when start/end markers are not present on the F2AC map; an approximate position is calculated as the distance between the start/end marker to the nearest F2AC mapped marker. Length (cM) is the centiMorgan distance between the F2AC map start and end of the change in marker order. Transverted map is the genetic map that shows marker order differences in the same map region with both other maps. Min start position and max end position are the smaller of two tranversion start positions and the larger of the two tranversion end positions, respectively, in the same genetic map region based on the two paired map comparisons.

**Figure 2.**
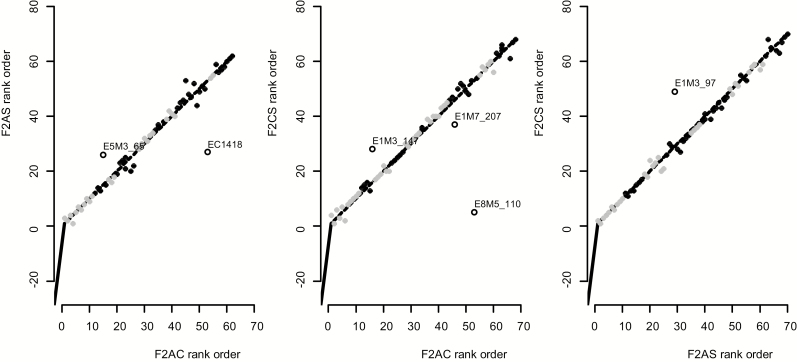
Paired comparisons of marker order between the F2AC, F2AS and F2CS genetic maps. Points indicate the relative map order of genetic markers common to each pair of compared genetic maps. Blocks of alternating light grey and black points indicate different LGs. Labelled circles indicate genetic markers in discrepant map positions (≥10 difference in relative map order) in each pair of compared genetic maps. The dashed lines indicate identical marker order for comparison. Summaries of Kendall’s rank correlation tests are shown in the top left corner of each panel.

### Transmission ratio distortion

Results of all TRD tests for genetic markers analysed in the F2AS and F2CS families are presented in **Supporting Information—Tables S2** and **S3**, respectively. Equivalent results for the F2AC family are available in Table S2 of Brennan *et al.* (2014). The three F_2_ mapping families differed significantly in the frequency of genetic markers showing genotypic TRD [Test 1, **see Supporting Information—Table S4**], with 26.8 %, 14 % and 2.9 % of markers exhibiting TRD in the F2AC, F2CS and F2AS mapping families, respectively. When genotypic TRD was tested using the more stringent map-wide 95 % confidence level, no difference was detected between the F2AC and F2CS mapping families in frequency of genetic markers showing TRD, but the difference between these two families and the F2AS mapping family remained, with the latter map containing no markers exhibiting genotypic TRD at this threshold **[see****Supporting Information—Table S4****]**. Unmapped genetic markers were more likely to show genotypic TRD in each mapping population.

**Figure 3.**
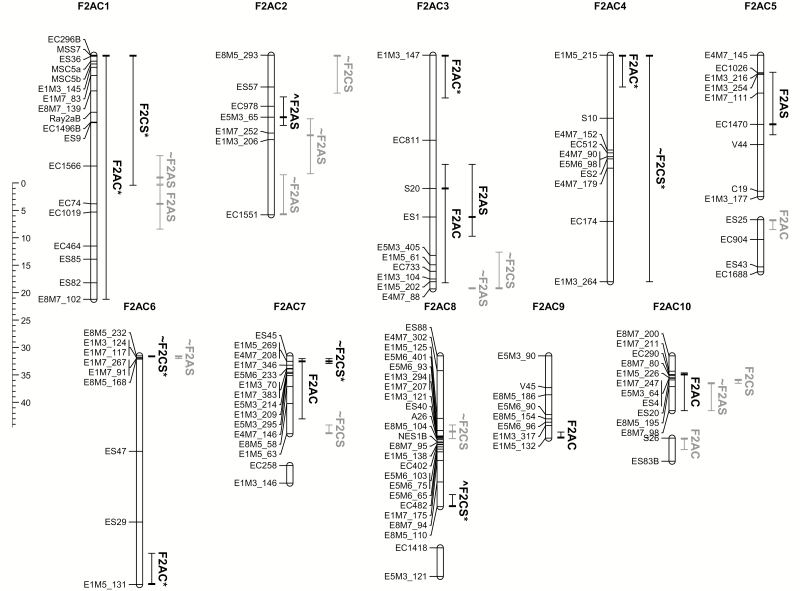
Genetic map locations and evidence for co-location of F2AS and F2CS transmission distortion loci transposed onto the F2AC genetic map. The F2AC genetic map was used as a reference against which to compare TRDL locations. Genetic map features are as described in the notes to Fig. 1. TRDL locations are represented as vertical lines to the right of F2AC LGs and named after the genetic map in which they were observed. The lines depicting TRDLs extend to cover linked genetic markers showing TRD. A bold cross hatch indicates the location of the marker with greatest TRD within that TRDL. Black lines indicate TRDLs identified from significantly biased genotype frequencies while grey lines indicate TRDLs identified by other tests (see Table 2). * after TRDL names indicates that χ^2^ test resulted in *P* < 0.0005. ~ before TRDL names indicates that the marker with greatest TRD was not found on the F2AC map but the approximate position and extent of the TRDL was estimated from synteny across genetic maps. ^ before TRDL names indicates that the marker with greatest TRD was present on a different LG compared to its F2AC location (see Table 2).

After combined markers with genotypic TRD into TRDLs, it was apparent that fewer TRDLs were detected in the F2AS and F2CS maps (containing four and six TRDLs, respectively) (Table 3, Fig. 3) than in the F2AC genetic map (containing nine TRDLs) (Table 3 in Brennan *et al.* 2014). Although most TRDLs identified in the F2AS and F2CS maps mapped to equivalent LGs in the F2AC genetic map, there were two notable exceptions. These included a TRDL represented by genetic marker E3M5_65, which mapped to LGs AC2 and AS4, and another represented by E8M5_110 that mapped to LGs AC8 and CS1. These exceptions could represent translocations or errors in mapping potentially caused by TRD itself.

**Table 3.** Summary of TRDLs observed for tests of different TRD mechanisms in the F2AS and F2CS genetic maps

Linkage group_map position (cM)	Equivalent AC LG and map position (cM)	Genetic marker with greatest TRD	Genotypic TRD	Asymmetric / cytonuclear TRD	Pre-zygotic TRD	Heterozygote deficit	Epistasis / BDMs (minority genotype)
AS2_3.7 singleton	AC2_9.3	EC978	Yes	No	No	Yes	No
AS4A_37.9 singleton	AC2_11.3	E5M3_65	Yes	Squal	–	–	No
AS3_0.0 cluster	AC3_29.5	ES1	Yes	Squal	No	Yes	No
AS5A_26.9 singleton	AC5A_12.6	EC1470	yes	Squal	No	–	No
CS1_6.6 cluster	AC1_0.0	EC296B	Yes	No	Squal	Yes	CS1_6.7 BC, CS4A_38.1 BD
CS4B_0.0 singleton	AC4A proximal	E5M3_219	Yes	No	–	–	No
CS4A_38.1 singleton	AC4A_41.3	E1M3_264	Yes	No	–	–	CS1_6.6 DB, CS1_6.7 CD
CS6A_0.0 singleton	AC6A proximal	E5M6_397	Yes	No	Yes	–	No
CS7A_unmapped	AC7A_1.0	E1M5_269	Yes	No	Yes	–	No
CS1_6.7 cluster	AC8A_27.5	E8M5_110	Yes	No	Yes	–	CS1_6.6 CB, CS4A_38.1 DC
AS1_28.4 singleton	AC1 central	E5M3_104	No	Aeth	–	–	–
AS2_29.7 singleton	AC2 central	ES56	No	Aeth	No	Yes	–
AS3_16.9 singleton	AC3 distal	ES58	No	Aeth	No	Yes	–
AS6_6.0 singleton	AC6 proximal	E1M5_140	No	Aeth	No	–	–
AS1_37.8 cluster	AC1_27.1	EC74	No	Squal	Squal	No	–
AS2_44.6 singleton	AC2 distal	ES74B	No	Squal	No	–	–
AS5A_0.0 cluster	AC5A_3.5	E1M3_254	No	Squal	–	–	–
AS10A_7.6 cluster	AC10A central	E8M7_223	No	Squal	No	–	–
CS4B_11.5 cluster	AC4A_18.9	ES2	No	Chrys	No	Yes	–
CS4B_67.2 singleton	AC4A distal	E1M8_106	No	Chrys	–	–	–
CS7A_13.5 singleton	AC7A distal	E8M7_226	No	Chrys	No	–	–
CS8A_6.8 singleton	AC8A central	E1M5_88	No	Both	–	–	–
CS10A_9.2 cluster	AC10A_4.4	ES20	No	Chrys	no	Hom	–
CS2_2.0 singleton	AC2 proximal	E1M3_163	No	Squal	–	–	–
CS3_21.7 cluster	AC3 distal	E5M6_187	No	Squal	No	–	–

Linkage group names correspond to Fig. 1. All tests for TRD involved χ^2^ tests against null expectations at a per-marker 95 % confidence level unless stated otherwise. ‘-’ indicates that genetic marker genotypes did not allow particular TRD tests. Cluster indicates that the TRDL is represented by multiple linked genetic markers less than 10 cM apart, whereas singleton indicates that the TRDL is represented by a single marker. Equivalent F2AC genetic map positions are estimated as ‘proximal’, ‘central’ or ‘distal’ based on additional linked genetic markers when the marker with greatest TRD was not itself present on the F2AC map. Genotypic TRD indicates markers that showed TRD for genotype frequencies. Asymmetric/cytonuclear TRD indicates if TRD was present for one parental cytotype only, showing the affected cytotype as ‘Aeth’ for *S. aethnensis*, ‘Chrys’ for *S. chrysanthemifolius* and ‘Squal’ for *S. squalidus*. Pre-zygotic TRD indicates if TRD was present for allelic frequencies showing the minority parental allele for significant cases. Heterozygote deficit indicates if a significant deficit of heterozygotes with both parental alleles was observed, with ‘Hom’ indicating the opposite case of a significant deficit of homozygotes. Epistasis / BDMs indicates if Fisher’s exact tests of paired genetic marker genotype combinations showed significant interactions indicative of two-locus BDM incompatibilities. Epistatic interactions are summarized as the other interacting TRDL locations and the minority genotype at the ‘home’ and ‘away’ TRDL. Genotypes are described in mapmaker format where ‘A’ and ‘B’ indicate homozygous parental alleles (parents listed alphabetically), ‘H’ indicates heterozygous parental alleles, ‘C’ indicates not homozygous A parent and ‘D’ indicates not homozygous B parent.

Further tests indicated the possible causes of TRD at a locus. Thus, Test 2 indicated examples of asymmetric TRD (i.e. dependent on cross direction) at three of four TRDLs resolved in the F2AS mapping family, but at none of the six loci resolved in the F2CS family (Table 3). Rather surprisingly, some additional asymmetric TRDLs (eight and four in the F2AS and F2CS maps, respectively) were detected by these cross-specific tests (Table 3, Fig. 3) that were not detected in the analysis of entire F2 progenies (Test 1).

Tests of pre-zygotic (or allelic) TRD (Test 3) were significant for one F2AS and four F2CS TRDLs (Table 3), whereas tests of parental allele heterozygote surplus or deficit (Test 4) showed deficits for one F2CS and two F2AS TRDLs resolved in the analyses of entire F2 families and two additional F2AS TRDLs resolved in the tests of asymmetric TRD (Table 3). Finally, tests of negative epistasis between TRDLs (Test 5) were significant for three interacting F2CS TRDLs represented by markers EC296B, E1M3_264 and E8M5_110 (Table 3). The minority genotype combinations for these TRDLs suggested a lack of *S. squalidus*-like genotypes. Two of these TRDLs, EC296B and E8M5_110, while on separate LGs in the F2CS map, form part of a large TRDL located on the AC1 LG of the F2AC map (Brennan *et al.* 2014).

### Co-location of TRDLs (genetic incompatibilities) across genetic maps and with genomic rearrangements

Comparison of the genomic distribution of TRDLs indicated that all LGs contained TRDLs across the three genetic maps with between one and five TRDLs observed per LG (Fig. 3, Table 3). There was evidence for co-location of TRDLs between genetic maps. In particular, the F2AC and F2CS genetic maps showed significantly more co-located TRDLs than expected by chance for all subsets of TRDLs and across a range of map interval test lengths from 2 to 8 cM **[see****Supporting Information—Figure S2****]**. Co-location tests at larger map interval lengths were probably more conservative as the random chance of multiple TRDLs occurring within the same large interval increased. Visual inspection of the maps identified these co-located TRDLs on LGs 1, 4, 7 and 10. No significant co-location of TRDLs was evident from comparisons of the F2AC and F2AS or the F2AS and F2CS maps. Co-location tests involving TRDLs that remained significant after correction for multiple testing found significant co-location of TRDLs on LGs 1 and 4 of the F2AC and F2CS maps **[see****Supporting Information—Figure S2**]. No evidence was found for TRDLs being co-located with the midpoints of genomic rearrangments as inclusion of these data into the co-location analyses did not change the significance of test statistics **[see****Supporting Information—Figure S2****]**.

## Discussion

The genetic mapping and TRD analyses conducted on F_2_ families generated from pairwise species crosses indicate that the homoploid hybrid species, *Senecio squalidus,* has inherited genetic incompatibilities from both of its parental species, *S. aethnensis* and *S. chrysanthemifolius*. These findings lend support to a model of how reproductive isolation of a homoploid hybrid species can be initiated by inheritance of pre-existing genetic incompatibilities between the parental species (Schumer *et al.* 2015). Although reproductive isolation of *S. squalidus* from its parents is primarily dependent on ecogeographic isolation, with some isolating effects possibly resulting from genetic drift or selection during its origin and establishment in the UK (James and Abbott 2005; Abbott *et al.* 2009; Ross 2010; Brennan *et al.* 2012), our results indicate that inherited genetic incompatibilities also contribute to the reproductive isolation of this homoploid hybrid species.

### Genetic maps

Our previous genetic mapping study using the same *S. aethnensis* and *S. chyrsanthemifolius* parental individuals as in this study suggested that the large-scale structure of the genomes of both species was similar with no genetic evidence of fusions, fissions or translocations among chromosomes (Brennan *et al.* 2014). This finding was further supported by the results of an independent genetic mapping study conducted by Chapman *et al.* (2016). The present investigation extended these analyses to a comparison of the genomic structure of *S. squalidus* with its progenitors and indicated that some large-scale genomic restructuring had occurred during the origin of *S. squalidus*, although the three genetic maps were very similar overall, in terms of number and length of LGs detected. Comparisons between the three genetic maps indicated that the 13 to 15 LGs present in each map could be assigned to the ten chromosomes expected for these *Senecio* species (Fig. 1 and **Supporting Information—Fig. S1**, Table 1). The maps also showed similar total and individual LG lengths, which corroborates the similar 2C nuclear DNA content measures of 1.57, 1.63 and 1.41 pg recorded for *S. aethnensis, S. chrysanthemifolius* and *S. squalidus*, respectively (Coyle and Abbott, unpubl. results). It seems, therefore, that genome size increase caused by retrotransposon proliferation activated by hybridization as reported in *Helianthus* (Baack *et al.* 2005; Ungerer *et al.* 2006) and *Aegilops* (Senerchia *et al.* 2016) is probably not a feature of this *Senecio* system.

### Nature of genomic rearrangements

Genetic mapping showed that approximately half of all component genetic markers were shared between each pair of genetic maps and these shared markers indicated high overall synteny between maps based on correlation tests (Fig. 2). A few genetic translocations between LGs, involving only one to three genetic markers that were found on non-equivalent LGs per paired map comparison, were observed (Fig. 2), and might reflect small-scale genomic translocations affecting individual genetic markers at a scale of 2 to 5 cM (one to two times the mean cM distance between mapped markers). However, an alternative explanation is that some of these genetic markers that map to different LGs in different genetic maps also show strong genotypic TRD in one or both genetic maps (E5M3_65 for the F2AC versus F2AS comparison and E1M7_207 and E8M5_110 for the F2AC versus F2CS comparison). Strong TRD could cause false associations between genotypes of unlinked markers leading to errors in map location.

Tests of marker order at the LG level found evidence for genomic transversions affecting at least five different genomic regions; one present on each of the F2AC (LG8) and F2AS (LG4) maps and three on LGs 3, 9 and 10 of the F2CS map (Fig. 1). These results suggest that the genomes of the three parental individuals representing each of the three study species are distinguished by one to three genomic rearrangements. Determining which rearrangement is associated with each species and which rearrangements might have been inherited by *S. squalidus* from its parental species would require additional genetic mapping studies of within species crosses. These rearranged genomic regions also show significantly shorter mapped marker distances than other genomic regions. This is because recombination is negatively selected within these genomic regions in the progeny of individuals heterozyogous for the rearrangement as it generates large deleterious insertion-deletion mutations (Fishman *et al.* 2013).

The genomic rearrangements identified could indicate genomic regions that potentially harbour many genes of functional significance that are protected from interspecific gene flow and upon which selection can act to promote divergent evolution. Genomic inversions have been found to be associated with multiple examples of divergent adaptive evolution in the presence of gene flow, for example, perennial and annual ecotypes of *Mimulus guttatus* (Twyford and Friedman 2015), and different Mullerian host mimics in the butterfly, *Heliconius numata* (Joron *et al.* 2011). It would be of interest to investigate further the potential contribution that genomic rearrangements between *S. aethnensis* and *S. chrysanthemifolius* make towards maintaining ecological differentiation despite gene flow across the Mount Etna hybrid zone. The interspecific genomic rearrangements observed in these genetic maps could also contribute to genetic incompatibility between *S. squalidus* and both of its parental species.

Genomic reconstruction during the origin of *S. squalidus* agrees with reports of the same in other homoploid hybrid species, e.g. in *Helianthus* (Burke *et al.* 2004) and in *Iris* (Tang *et al.* 2010; Taylor *et al.* 2013). In these cases, genomic reorganization was interpreted as a ‘genomic shock’ response to hybridization (McClintock 1984; Rieseberg 2001; Chen and Ni 2006), enabling stabilization of the hybrid genome, and associated positively with evolutionary divergence of the parental genomes. The parental species of *S. squalidus* are estimated to have diverged relatively recently, around 150 000 years ago, once suitable habitats for the high altitude species *S. aethnensis* became available with the rise of the volcano, Mount Etna, in Sicily (Chapman *et al.* 2013; Osborne *et al.* 2013). Despite their very recent origin, some genomic rearrangements appear to have already emerged between these two species. In combination with findings for other homoploid hybrid species (Tang *et al.* 2010; Taylor *et al.* 2013), our results suggest that genomic restructuring is a frequent feature of the successful establishment of new hybrid species in combination with ecological and/or spatial divergence from parental species (Buerkle *et al.* 2000; Baack and Rieseberg 2007, Karrenberg *et al.* 2007).

### Transmission ratio distortion

The presence of TRD among genotyped progenies is indicative of genetic incompatibilities between the parental lines because particular alleles, genotypes, or combinations of these have been selected against in hybrid offspring (Fishman *et al.* 2001; Harushima *et al.* 2001; Hall and Willis 2005; Moyle and Graham 2006). Comparisons between genetic maps showed that TRD was present at multiple genomic regions across all chromosomes. Most genotype-level TRDLs and the highest proportion of genetic markers exhibiting TRD were evident in the F2AC map (Fig. 3, Table 3 and **Supporting Information—Table S4**). This observation fits with evidence that hybrid incompatibilities have accumulated and been reinforced by divergent ecological selection between the parental species on Mount Etna (Osborne *et al.* 2013; Brennan *et al.* 2014; Chapman *et al.* 2016; Filatov *et al.* 2016), in contrast to incompatibilities involving the hybrid species that no longer interacts with its parents due to geographic isolation.

The crossing design used to produce the F_2_ mapping families involved full-sib F_1_ crosses raising the possibility that some of the observed TRD could be due to bi-parental inbreeding. These species are self-incompatible and *S. squalidus* has been shown to suffer from inbreeding depression when selfed (Brennan *et al.* 2005, 2013). Inbreeding depression would affect patterns of TRD in the form of selection against reconstituted homozygous F_0_ parental genotypes in the F_2_ progeny. However, at TRDLs where tests of TRD of heterozygosity of marker alleles of different F_0_ parental origin could be applied, the majority of significant results were in favour of a deficit of heterozygotes (one out of one tests for the F2AC map, four out of four tests for the F2AS map, two out of three tests for the F2CS map, Table 3). Therefore, most TRDLs in these mapping families appear to be caused by genetic incompatibilities between species rather than inbreeding depression.

Less TRD was evident in the F_2_ families produced from crosses between each parental species and *S. squalidus* than between the two parent species. For the families involving *S. squalidus*, 14 % of markers were distributed across six TRDLs in the F2CS family compared with 2.9 % of markers showing TRD across four TRDLs in the F2AS family (**Supporting Information—Table S4**). The extent of genotypic TRD was also more pronounced for many F2CS TRDLs relative to F2AS TRDLs, indicating that *S. squalidus* inherited a greater number of *S. aethnensis*-like incompatibility alleles or local rearrangements that preferentially cause genetic incompatibility with the *S. chrysanthemifolius* parent. Asymmetric backcross incompatibility and directions of introgression have been reported for a number of hybridizing species and can be caused by cytoplasmic incompatibilities between nuclear and chloroplastic genomes (Buerkle and Rieseberg 2001; Martin *et al.* 2005; Scascitelli *et al.* 2010; Senerchia *et al.* 2016; Abbott 2017). In the case of *S. squalidus,* samples have been found to share the same chloroplast DNA haplotype with both parental species, so the direction of the original hybrid cross is currently uncertain (Abbott *et al.* 1995; Comes and Abbott 2001; Simon Hiscock, unpubl. data). The TRD tests that took cytoplasmic identity into account (Test 2) found only two instances of asymmetric TRD in each of the F2AS and F2CS mapping families, suggesting that cytoplasmic incompabilities are minor contributors to the overall hybrid incompatibility observed in this system and supporting the hypothesis that hybridization in both cross directions could contribute to gene flow and hybrid evolution.

The greater prevalence of genetic incompatibilities between *S. squalidus* and *S. chrysanthemifolius* does not appear to have biased parental contributions to the hybrid genome of *S. squalidus* (James and Abbott 2005; Brennan *et al.* 2012; Filatov *et al.* 2016). Instead, the effect of these genetic incompatibilities on hybridization dynamics would seem to be restricted to smaller genomic regions. Considering all the forms of TRD identified, each cross showed multiple TRDLs distributed across the genome that function in a mixture of cross directions, so that their combined effect would contribute to genetic incompatibility in both cross directions.

Similar to results previously reported for the F2AC mapping family (Brennan *et al.* 2014), additional tests of TRD provided evidence for cytonuclear incompatibilities, allelic pre-zygotic incompatibilities, heterozygote (and homozygote) deficit and two-locus epistatic incompatibilities as causes of TRD in both F2AS and F2CS mapping families (Table 3). Moreover, neighbouring TRDLs (<10 cM apart) were identified to exhibit TRD resulting from different causes, as demonstrated for the neighbouring pairs of F2AS TRDLs on LGs 1 and 2 (Fig. 3). It seems likely, therefore, that additional TRDLs would be detected if TRD in hybrid crosses were to be studied at greater resolution with more markers employed. Insufficient genomic resolution might, in part, explain why some TRDLs were observed in only one family out of the three studied. It needs emphasizing that the construction of these genetic maps involved a single representative of each species and therefore represents a snapshot of all the genetic incompatibilities that are present in this system. Genetic maps built from different parents might reveal a slightly different subset of genetic incompatibilities if the alleles causing these incompatiblities have not been fixed in the different species, as noted in hybridizing *Mimulus guttatus* and *M. nasutus* (Sweigart *et al.* 2007; Martin and Willis 2010). It is less likely, but not inconceiveable, that new genetic incompatibilities such as BDMs could have emerged between the parental species and *S. squalidus* since it became allopatrically isolated in the UK approximately 320 generations ago.

The results of this study confirm that, in addition to previously identified intrinsic hybrid incompatibilities between *S. aethnensis* and *S. chrysanthemifolius* (Brennan *et al.* 2009, 2014; Chapman *et al.* 2016), genetic incompatibilities are also present between the homoploid hybrid species, *S. squalidus*, and its parental species. We also found support for the hypothesis that these genetic incompatibilities in *S. squalidus* were inherited from its progenitors by testing for genetic incompatibilities that were shared between the different F_2_ mapping families. TRDLs were found to be significantly co-located between the F2AC and F2CS maps based on four co-located TRDLs on LGs 1, 4, 7 and 10 (Fig. 3). Taken overall, the evidence we obtained of multiple shared TRDLs in the genetic maps of crosses between the hybrid species and its progenitors supports the evolutionary potential of inheritance and re-assortment of hybrid incompatibilities (Grant 1981; Paixão *et al.* 2014; Schumer *et al.* 2015b).

## Conclusions

While evidence for the contribution of hybridization to speciation continues to accumulate in the literature (Abbott 2017 and references therein), our understanding of the process at a genomic level is still very limited. Our study addresses this knowledge gap using a genetic mapping approach to investigate the structure of the genome of a new homoploid hybrid species in comparison to its progenitors. Our results reinforce the view that hybridization has heterogeneous effects across the genome at multiple dispersed genomic locations. A challenge for the future is to examine a greater variety of naturally hybridizing systems at a sufficiently dense genomic resolution to determine the generality of these observations and to zoom in on the particular genes or genomic structures acting as hybridizing barriers (e.g. Christe *et al.* 2017). There continues to be a need to integrate new genetic data with data on the effects of hybridization on quantitative traits and fitness, particularly in the environments where hybridization actually occurs (Goulet *et al.* 2017). The developing applicability of high-throughput sequencing methods and their analysis to non-model hybridizing systems will contribute to these issues and provide new insights into the evolutionary consequences of hybridization.

## Data archiving

Mapping family genotype data and genetic map information has been deposited with the DRYAD Digital Repository (https://doi.org/10.5061/dryad.82d5f33). Other results can be found in the supporting information.

## Source of Funding

The research was funded by an Natural Environment Research Council (grant NE/D014166/1) to RJA as Principal Investigator.

## Contributions by the Authors

A.C.B., S.J.H. and R.J.A. conceived the study. A.C.B. performed the experiments and analysed the results. A.C.B., S.J.H. and R.J.A. contributed to writing the paper.

## Conflict of Interest

None declared.

## Supplementary Material

Supplementary Table S1

Supplementary Table S2

Supplementary Table S3

Supplementary Table S4

Supplementary Table S5

Supplementary Figures S1 and S2

## Supporting Information

The following additional information is available in the online version of this article:

Table S1. Summary of codominant molecular markers used for mapping.

Table S2. Frequencies and segregation tests of genetic markers genotyped in the F2AS mapping family.

Table S3. Frequencies and segregation tests of genetic markers genotyped in the F2CS mapping family.

Table S4. Summary of genetic markers showing TRD on the F2AC, F2AS and F2CS genetic maps.

Table S5. Summary of tests for co-location of TRDLs and rearrangements for each paired genetic map comparison.

Figure S1. Interleaved genetic maps of all linkage groups from the F2AC, F2AS and F2CS mapping families.

Figure S2. Probability of co-location of TRD loci and rearranged genomic regions for each paired genetic map comparison across a range of genomic size intervals.

## References

[CIT0001] Abbott RJ 2017 Plant speciation across environmental gradients and the occurrence and nature of hybrid zones. Journal of Evolutionary Biology 55:238–258.

[CIT0002] Abbott R, Albach D, Ansell S, Arntzen JW, Baird SJ, Bierne N, Boughman J, Brelsford A, Buerkle CA, Buggs R, Butlin RK, Dieckmann U, Eroukhmanoff F, Grill A, Cahan SH, Hermansen JS, Hewitt G, Hudson AG, Jiggins C, Jones J, Keller B, Marczewski T, Mallet J, Martinez-Rodriguez P, Möst M, Mullen S, Nichols R, Nolte AW, Parisod C, Pfennig K, Rice AM, Ritchie MG, Seifert B, Smadja CM, Stelkens R, Szymura JM, Väinölä R, Wolf JB, Zinner D 2013 Hybridization and speciation. Journal of Evolutionary Biology 26:229–246. 23323997 10.1111/j.1420-9101.2012.02599.x

[CIT0003] Abbott RJ, Brennan AC, James JK, Forbes DG, Hegarty MJ, Hiscock SJ 2009 Recent hybrid origin and invasion of the British Isles by a self-incompatible species, Oxford ragwort (*Senecio squalidus* L., Asteraceae). Biological Invasions 11:1145–1158.

[CIT0005] Abbott RJ, Curnow DJ, Irwin JA 1995 Molecular systematics of *Senecio squalidus* L. and its close diploid relatives. In: Hind DJN, Jeffrey C, Pope GV, eds. Advances in compositae systematics. Kew: Royal Botanic Gardens, 223–237.

[CIT0006] Alexander JCM 1979 Mediterranean species of *Senecio* sections *Senecio* and *Delphinifolius*. Notes from the Royal Botanic Garden, Edinburgh 37:387–428.

[CIT0007] Allan E, Pannell JR 2009 Rapid divergence in physiological and life‐history traits between northern and southern populations of the British introduced neo‐species, *Senecio squalidus*. Oikos 118:1053–1061.

[CIT0008] Baack EJ, Rieseberg LH 2007 A genomic view of introgression and hybrid speciation. Current Opinion in Genetics & Development 17:513–518. 17933508 10.1016/j.gde.2007.09.001 PMC2173880

[CIT0009] Baack EJ, Whitney KD, Rieseberg LH 2005 Hybridization and genome size evolution: timing and magnitude of nuclear DNA content increases in *Helianthus* homoploid hybrid species. The New Phytologist 167:623–630. 15998412 10.1111/j.1469-8137.2005.01433 PMC2442926

[CIT0010] Brennan AC, Barker D, Hiscock SJ, Abbott RJ 2012 Molecular genetic and quantitative trait divergence associated with recent homoploid hybrid speciation: a study of *Senecio squalidus* (Asteraceae). Heredity 108:87–95. 21829224 10.1038/hdy.2011.46 PMC3262868

[CIT0011] Brennan AC, Bridle JR, Wang AL, Hiscock SJ, Abbott RJ 2009 Adaptation and selection in the *Senecio* (Asteraceae) hybrid zone on Mount Etna, Sicily. The New Phytologist 183:702–717. 19594693 10.1111/j.1469-8137.2009.02944.x

[CIT0012] Brennan AC, Harris SA, Hiscock SJ 2005 Modes and rates of selfing and associated inbreeding depression in the self-incompatible plant *Senecio squalidus* (Asteraceae): a successful colonizing species in the British Isles. The New Phytologist 168:475–486. 16219086 10.1111/j.1469-8137.2005.01517.x

[CIT0012a] Brennan AC, Harris SA, Hiscock SJ 2013 The population genetics of sporophytic self-incompatibility in three hybridizing *Senecio* (Asteraceae) species with contrasting population histories. Evolution 67:1347–1367. 23617913 10.1111/evo.12033

[CIT0013] Brennan AC, Hiscock SJ, Abbott RJ 2014 Interspecific crossing and genetic mapping reveal intrinsic genomic incompatibility between two *Senecio* species that form a hybrid zone on Mount Etna, Sicily. Heredity 113:195–204. 24595365 10.1038/hdy.2014.14 PMC4815642

[CIT0014] Brennan AC, Hiscock SJ, Abbott RJ 2016 Genomic architecture of phenotypic divergence between two hybridizing plant species along an elevational gradient. AoB PLANTS 8: doi:10.1093/aobpla/plw022. 10.1093/aobpla/plw022 PMC4887755 27083198

[CIT0015] Buerkle CA, Morris RJ, Asmussen MA, Rieseberg LH 2000 The likelihood of homoploid hybrid speciation. Heredity 84:441–451. 10849068 10.1046/j.1365-2540.2000.00680.x

[CIT0016] Buerkle CA, Rieseberg LH 2001 Low intraspecific variation for genomic isolation between hybridizing sunflower species. Evolution; International Journal of Organic Evolution 55:684–691. 11392386 10.1554/0014-3820(2001)055[0684:livfgi]2.0.co;2

[CIT0017] Burke JM, Lai Z, Salmaso M, Nakazato T, Tang S, Heesacker A, Knapp SJ, Rieseberg LH 2004 Comparative mapping and rapid karyotypic evolution in the genus *Helianthus*. Genetics 167:449–457. 15166168 10.1534/genetics.167.1.449 PMC1470840

[CIT0018] Chakravarti A, Lasher LK, Reefer JE 1991 A maximum likelihood method for estimating genome length using genetic linkage data. Genetics 128:175–182. 2060775 10.1093/genetics/128.1.175 PMC1204446

[CIT0019] Chapman MA, Hiscock SJ, Filatov DA 2013 Genomic divergence during speciation driven by adaptation to altitude. Molecular Biology and Evolution 25:2467–2481. 10.1093/molbev/mst168 PMC3840311 24077768

[CIT0020] Chapman MA, Hiscock SJ, Filatov DA 2016 The genomic bases of morphological divergence and reproductive isolation driven by ecological speciation in *Senecio* (Asteraceae). Journal of Evolutionary Biology 29:98–113. 26414668 10.1111/jeb.12765

[CIT0021] Chen ZJ, Ni Z 2006 Mechanisms of genomic rearrangements and gene expression changes in plant polyploids. Bioessays: News and Reviews in Molecular, Cellular and Developmental Biology 28:240–252. 10.1002/bies.20374 PMC1986666 16479580

[CIT0022] Christe C, Stölting KN, Paris M, Fraїsse C, Bierne N, Lexer C 2017 Adaptive evolution and segregating load contribute to the genomic landscape of divergence in two tree species connected by episodic gene flow. Molecular Ecology 26:59–76. 27447453 10.1111/mec.13765

[CIT0004] Comes HP, Abbott RJ 2001 Molecular phylogeography, reticulation, and lineage sorting in Mediterranean *Senecio* sect. *Senecio* (Asteraceae). Evolution 55:1943–1962. 11761056

[CIT0023] Coyne JA, Orr HA 2004 Speciation. Sunderland, MA: Sinaeur Associates.

[CIT0023a] Duenez-Guzman EA, Mavárez J, Vose MD, Gavrilets S 2009 Case studies and mathematical models of ecological speciation. 4. Hybrid speciation in butterflies in a jungle. Evolution 63:2611–2626. 19545268 10.1111/j.1558-5646.2009.00756.x

[CIT0024] Filatov DA, Osborne OG, Papadopulos AS 2016 Demographic history of speciation in a senecio altitudinal hybrid zone on Mt. Etna. Molecular Ecology 25:2467–2481. 26994342 10.1111/mec.13618

[CIT0025] Fishman L, Kelly AJ, Morgan E, Willis JH 2001 A genetic map in the *Mimulus guttatus* species complex reveals transmission ratio distortion due to heterospecific interactions. Genetics 159:1701–1716. 11779808 10.1093/genetics/159.4.1701 PMC1461909

[CIT0026] Fishman L, Stathos A, Beardsley PM, Williams CF, Hill JP 2013 Chromosomal rearrangements and the genetics of reproductive barriers in *Mimulus* (monkey flowers). Evolution; International Journal of Organic Evolution 67:2547–2560. 24033166 10.1111/evo.12154

[CIT0027] Goulet BE, Roda F, Hopkins R 2017 Hybridization in plants: old ideas, new techniques. Plant Physiology 173:65–78. 27895205 10.1104/pp.16.01340 PMC5210733

[CIT0028] Grant V 1981 Plant speciation, 2nd edn. New York: Columbia University Press.

[CIT0029] Hall MC, Willis JH 2005 Transmission ratio distortion in intraspecific hybrids of *Mimulus guttatus*: implications for genomic divergence. Genetics 170:375–386. 15781698 10.1534/genetics.104.038653 PMC1449724

[CIT0030] Harushima Y, Nakagahra M, Yano M, Sasaki T, Kurata N 2001 A genome-wide survey of reproductive barriers in an intraspecific hybrid. Genetics 159:883–892. 11606560 10.1093/genetics/159.2.883 PMC1461833

[CIT0031] Hegarty MJ, Barker GL, Brennan AC, Edwards KJ, Abbott RJ, Hiscock SJ 2008 Changes to gene expression associated with hybrid speciation in plants: further insights from transcriptomic studies in *Senecio*. Philosophical Transactions of the Royal Society of London. Series B, Biological Sciences 363:3055–3069. 18579474 10.1098/rstb.2008.0080 PMC2607317

[CIT0032] Hiscock SJ 2000 Genetic control of self-incompatibility in *Senecio squalidus* L. (Asteraceae): a successful colonizing species. Heredity 85:10–19. 10971686 10.1046/j.1365-2540.2000.00692.x

[CIT0033] James JK, Abbott RJ 2005 Recent, allopatric, homoploid hybrid speciation: the origin of *Senecio squalidus* (Asteraceae) in the British Isles from a hybrid zone on Mount Etna, Sicily. Evolution; International Journal of Organic Evolution 59:2533–2547. 16526502

[CIT0034] Jiggins CD, Salazar C, Linares M, Mavarez J 2008 Hybrid trait speciation and *Heliconius* butterflies. Philosophical Transactions of the Royal Society of London. Series B, Biological Sciences 363:3047–3054. 18579480 10.1098/rstb.2008.0065 PMC2607310

[CIT0035] Joron M, Frezal L, Jones RT, Chamberlain NL, Lee SF, Haag CR, Whibley A, Becuwe M, Baxter SW, Ferguson L, Wilkinson PA, Salazar C, Davidson C, Clark R, Quail MA, Beasley H, Glithero R, Lloyd C, Sims S, Jones MC, Rogers J, Jiggins CD, ffrench-Constant RH 2011 Chromosomal rearrangements maintain a polymorphic supergene controlling butterfly mimicry. Nature 477:203–206. 21841803 10.1038/nature10341 PMC3717454

[CIT0036] Karrenberg S, Lexer C, Rieseberg LH 2007 Reconstructing the history of selection during homoploid hybrid speciation. The American Naturalist 169:725–737. 10.1086/516758 PMC2442913 17479459

[CIT0037] Lai Z, Nakazato T, Salmaso M, Burke JM, Tang S, Knapp SJ, Rieseberg LH 2005 Extensive chromosomal repatterning and the evolution of sterility barriers in hybrid sunflower species. Genetics 171:291–303. 16183908 10.1534/genetics.105.042242 PMC1456521

[CIT0038] Lexer C, Welch ME, Raymond O, Rieseberg LH 2003 The origin of ecological divergence in *Helianthus paradoxus* (Asteraceae): selection on transgressive characters in a novel hybrid habitat. Evolution; International Journal of Organic Evolution 57:1989–2000. 14575321 10.1111/j.0014-3820.2003.tb00379.x

[CIT0039] Lindtke D, Buerkle CA 2015 The genetic architecture of hybrid incompatibilities and their effect on barriers to introgression in secondary contact. Evolution; International Journal of Organic Evolution 69:1987–2004. 26174368 10.1111/evo.12725

[CIT0040] Mable BK, Alexandrou MA, Taylor MI 2011 Genome duplication in amphibians and fish: an extended synthesis. Journal of Zoology 284:151–182.

[CIT0041] Mallet J 2005 Hybridization as an invasion of the genome. Trends in Ecology & Evolution 20:229–237. 16701374 10.1016/j.tree.2005.02.010

[CIT0042] Marques I, Jürgens A, Aguilar JF, Feliner GN 2016 Convergent recruitment of new pollinators is triggered by independent hybridization events in *Narcissus*. The New Phytologist 210:731–742. 26738752 10.1111/nph.13805

[CIT0043] Martin NH, Bouck AC, Arnold ML 2005 Loci affecting long-term hybrid survivorship in Louisiana irises: implications for reproductive isolation and introgression. Evolution; International Journal of Organic Evolution 59:2116–2124. 16405157

[CIT0044] Martin NH, Willis JH 2010 Geographical variation in postzygotic isolation and its genetic basis within and between two *Mimulus* species. Philosophical Transactions of the Royal Society of London. Series B, Biological Sciences 365:2469–2478. 20643736 10.1098/rstb.2010.0030 PMC2935099

[CIT0044a] Mavárez J, Salazar CA, Bermingham E, Salcedo C, Jiggins CD, Linares M 2006 Speciation by hybridization in *Heliconius* butterflies. Nature 441:868–871. 16778888 10.1038/nature04738

[CIT0045] McCarthy EM, Asmussen MA, Anderson WW 1995 A theoretical assessment of recombinational speciation. Heredity 74:502–509.

[CIT0046] McClintock B 1984 The significance of responses of the genome to challenge. Science (New York, N.Y.) 226:792–801. 10.1126/science.15739260 15739260

[CIT0047] Moyle LC, Graham EB 2006 Genome-wide associations between hybrid sterility QTL and marker transmission ratio distortion. Molecular Biology and Evolution 23:973–980. 16495346 10.1093/molbev/msj112

[CIT0048] Nieto Feliner G, Álvarez I, Fuertes-Aguilar J, Heuertz M, Marques I, Moharrek F, Piñeiro R, Riina R, Rosselló JA, Soltis PS, Villa-Machío I 2017 Is homoploid hybrid speciation that rare? An empiricist’s view. Heredity 118:513–516. 28295029 10.1038/hdy.2017.7 PMC5436029

[CIT0049] Orr HA, Presgraves DC 2000 Speciation by postzygotic isolation: forces, genes and molecules. Bioessays: News and Reviews in Molecular, Cellular and Developmental Biology 22:1085–1094. 10.1002/1521-1878(200012)22:12<1085::AID-BIES6>3.0.CO;2-G 11084624

[CIT0050] Osborne OG, Batstone TE, Hiscock SJ, Filatov DA 2013 Rapid speciation with gene flow following the formation of Mt. Etna. Genome Biology and Evolution 5:1704–1715. 23973865 10.1093/gbe/evt127 PMC3787679

[CIT0051] Paixão T, Bassler KE, Azevedo RB 2014 Emergent speciation by multiple Dobzhansky-Muller incompatibilities. bioRxiv. doi:10.1101/008268

[CIT0052] Presgraves DC 2010 The molecular evolutionary basis of species formation. Nature Reviews. Genetics 11:175–180. 10.1038/nrg2718 20051985

[CIT0053] R Development Core Team 2017 R: a language and environment for statistical computing. Vienna, Austria: R Foundation for Statistical Computing.

[CIT0054] Rieseberg LH 2001 Chromosomal rearrangements and speciation. Trends in Ecology & Evolution 16:351–358. 11403867 10.1016/s0169-5347(01)02187-5

[CIT0055] Rieseberg LH, Willis JH 2007 Plant speciation. Science (New York, N.Y.) 317:910–914. 10.1126/science.1137729 PMC2442920 17702935

[CIT0056] Ross RI 2010 Local adaptation and adaptive divergence in a hybrid species complex in *Senecio*. DPhil Thesis, University of Oxford.

[CIT0057] Ross RI, Agren JA, Pannell JR 2012 Exogenous selection shapes germination behaviour and seedling traits of populations at different altitudes in a *Senecio* hybrid zone. Annals of Botany 110:1439–1447. 23071216 10.1093/aob/mcs211 PMC3489152

[CIT0058] Scascitelli M, Whitney KD, Randell RA, King M, Buerkle CA, Rieseberg LH 2010 Genome scan of hybridizing sunflowers from texas (*Helianthus annuus* and *H. Debilis*) reveals asymmetric patterns of introgression and small islands of genomic differentiation. Molecular Ecology 19:521–541. 20355258 10.1111/j.1365-294x.2009.04504.x

[CIT0059] Schumer M, Cui R, Powell DL, Dresner R, Rosenthal GG, Andolfatto P 2014a High-resolution mapping reveals hundreds of genetic incompatibilities in hybridizing fish species. eLife 3:e02535. 10.7554/eLife.02535 PMC4080447 24898754

[CIT0060] Schumer M, Cui R, Rosenthal GG, Andolfatto P 2015 Reproductive isolation of hybrid populations driven by genetic incompatibilities. PLoS Genetics 11:e1005041. 25768654 10.1371/journal.pgen.1005041 PMC4359097

[CIT0061] Schumer M, Rosenthal GG, Andolfatto P 2014b How common is homoploid hybrid speciation? Evolution; International Journal of Organic Evolution 68:1553–1560. 24620775 10.1111/evo.12399

[CIT0062] Schwarz D, Matta BM, Shakir-Botteri NL, McPheron BA 2005 Host shift to an invasive plant triggers rapid animal hybrid speciation. Nature 436:546–549. 16049486 10.1038/nature03800

[CIT0063] Senerchia N, Felber F, North B, Sarr A, Guadagnuolo R, Parisod C 2016 Differential introgression and reorganization of retrotransposons in hybrid zones between wild wheats. Molecular Ecology 25:2518–2528. 26678573 10.1111/mec.13515

[CIT0064] Servedio MR, Van Doorn GS, Kopp M, Frame AM, Nosil P 2011 Magic traits in speciation: ‘magic’ but not rare? Trends in Ecology & Evolution 26:389–397. 21592615 10.1016/j.tree.2011.04.005

[CIT0065] Soltis DE, Visger CJ, Soltis PS 2014 The polyploidy revolution then…and now: stebbins revisited. American Journal of Botany 101:1057–1078. 25049267 10.3732/ajb.1400178

[CIT0066] Stelkens R, Seehausen O 2009 Genetic distance between species predicts novel trait expression in their hybrids. Evolution; International Journal of Organic Evolution 63:884–897. 19220450 10.1111/j.1558-5646.2008.00599.x

[CIT0067] Sweigart AL, Mason AR, Willis JH 2007 Natural variation for a hybrid incompatibility between two species of *Mimulus*. Evolution; International Journal of Organic Evolution 61:141–151. 17300433 10.1111/j.1558-5646.2007.00011.x

[CIT0068] Tang S, Okashah RA, Knapp SJ, Arnold ML, Martin NH 2010 Transmission ratio distortion results in asymmetric introgression in Louisiana iris. BMC Plant Biology 10:48. 20298609 10.1186/1471-2229-10-48 PMC2923522

[CIT0069] Taylor SJ, Rojas LD, Ho SW, Martin NH 2013 Genomic collinearity and the genetic architecture of floral differences between the homoploid hybrid species *Iris nelsonii* and one of its progenitors, *Iris hexagona*. Heredity 110:63–70. 23047202 10.1038/hdy.2012.62 PMC3522232

[CIT0070] Twyford AD, Friedman J 2015 Adaptive divergence in the monkey flower *Mimulus guttatus* is maintained by a chromosomal inversion. Evolution; International Journal of Organic Evolution 69:1476–1486. 25879251 10.1111/evo.12663 PMC5029580

[CIT0071] Ungerer MC, Strakosh SC, Zhen Y 2006 Genome expansion in three hybrid sunflower species is associated with retrotransposon proliferation. Current Biology: CB 16:R872–R873. 17055967 10.1016/j.cub.2006.09.020

[CIT0072] Van Ooijen JW 2001 JoinMap v3.0 software for the calculation of genetic linkage maps Wageningen: Plant Research International http://www.joinmap.nl

[CIT0073] Yakimowski SB, Rieseberg LH 2014 The role of homoploid hybridization in evolution: a century of studies synthesizing genetics and ecology. American Journal of Botany 101:1247–1258. 25156978 10.3732/ajb.1400201

