# Supplementary material for: Completing the hybridization triangle: the inheritance of genetic incompatibilities during homoploid hybrid speciation in ragworts (Senecio)
Source: AoB Plants. 2019 Jan 6;11(1):ply078. doi: 10.1093/aobpla/ply078 (PMC6360072; doi:10.1093/aobpla/ply078)
Supplement: Supplementary Figures S1 and S2 [file ply078_suppl_supplementary_figures-s1-s2.docx]

Figure S1. Interleaved genetic maps of all linkage groups from the F2AC, F2AS, and F2CS mapping families. Legend to Figure S1. Map distances in Kosambi centiMorgans are shown in the scale to the left of linkage groups. Linkage groups (LGs) are represented by vertical bars with mapped marker positions indicated with horizontal lines. Linkage group names are presented in bold above each LG with letters indicating the pair of F_0_ species; *S. aethnensis* (A), *S. chrysanthemifolius* (C), or *S. squalidus* (S) that founded the mapping family and numbers indicating equivalent linkage groups that share genetic markers across the three maps. Weakly linked LGs that are thought to belong to the same chromosome are aligned vertically under a single overall LG name. Marker names are listed to the left of LGs in grey if they are common to another genetic map or in black if they are uniquely present on that genetic map. Dotted lines link common marker positions on the equivalent LGs of different genetic maps. Black shaded portions of LGs indicate chromosomal transversions identified from switches in marker order compared to equivalent LGs.

| 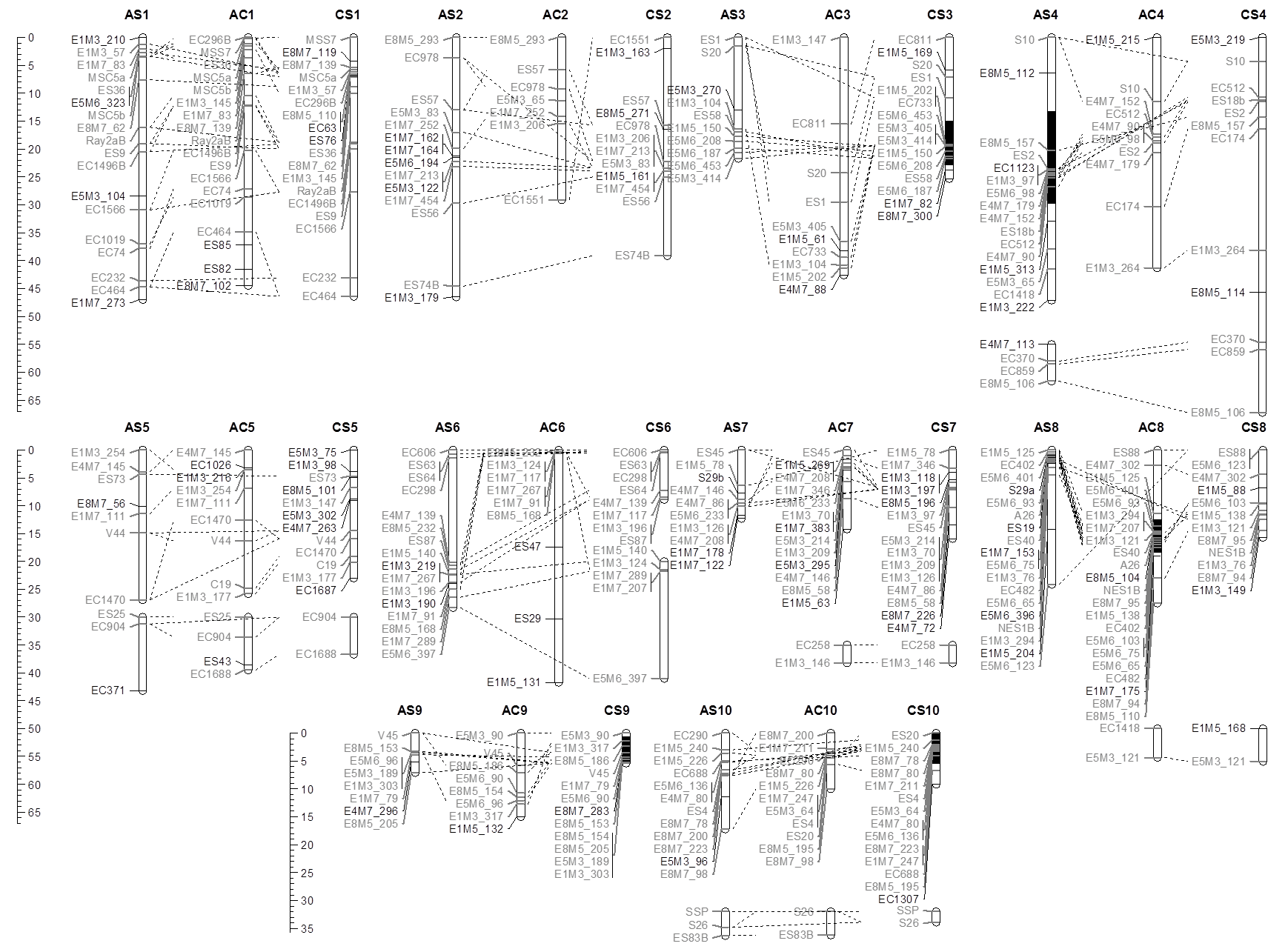 |
| --- |

Figure S2. Probability of colocation of transmission ratio distortion loci and rearranged genomic regions for each paired genetic map comparison across a range of genomic size intervals. Legend to figure S2. The genomic features being tested for colocation are shown in the titles to each panel. The influence of testing different genetic map interval lengths is shown on the x axis. The probability of colocation is shown on the y axis. The change in probability of colocation for each paired map comparison when testing different map interval sizes is indicated by different hatched lines as shown in the central legend. The horizontal grey dashed line indicates the *p* = 0.05 significance threshold, below which co-location of genomic features between genetic maps at a particular map interval resolution is supported. Lines for the F2AC versus F2AS and F2AS versus F2CS maps are missing for the central panel showing colocation probabilities of TRDs with map-level support at a 95 % confidence level because no TRDs were detected on the F2AS map at this threshold.

| 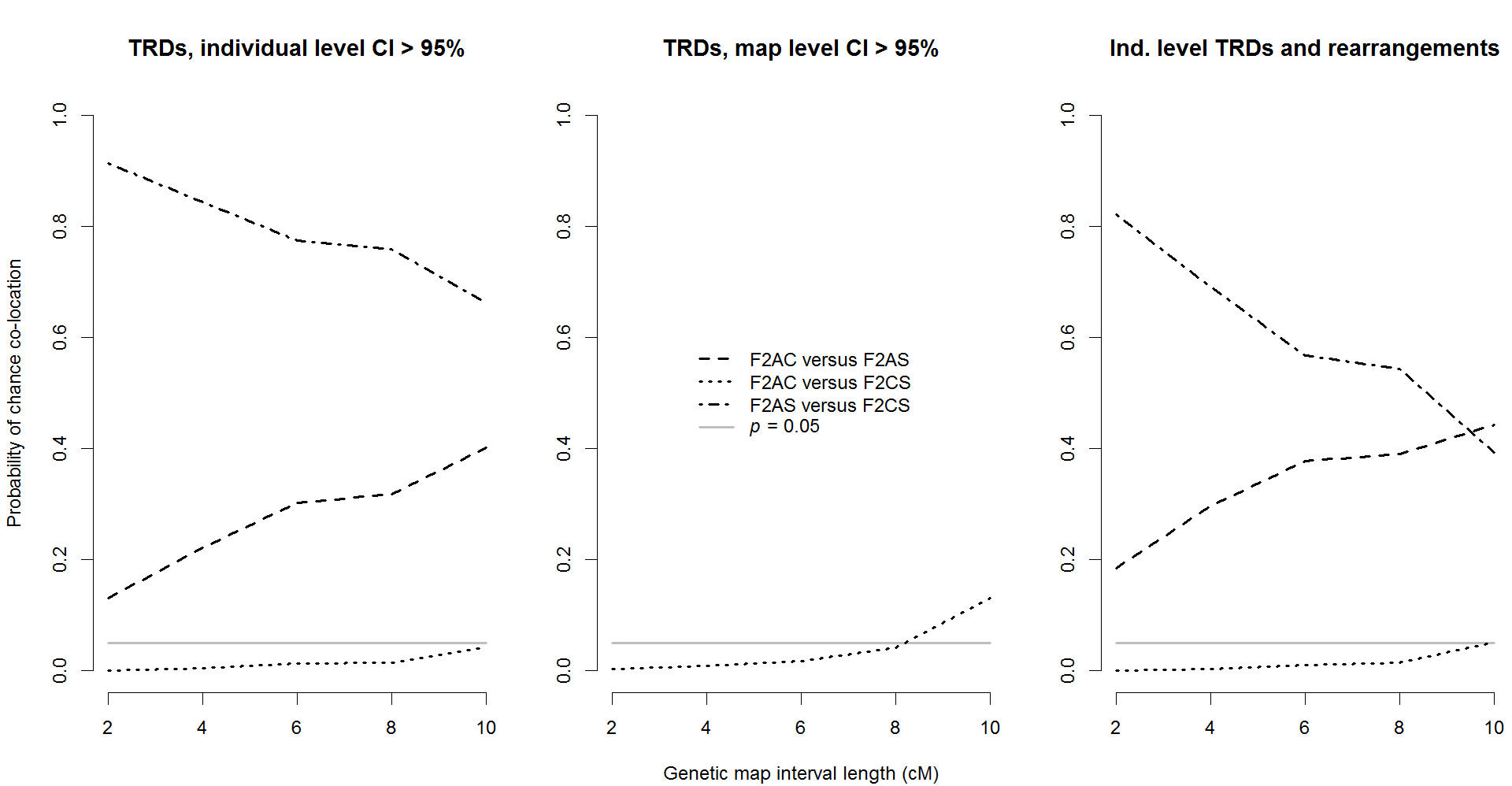 |
| --- |
